# Bronchoscopy of Lung Cancer

**DOI:** 10.1155/DTE.1.9

**Published:** 1994

**Authors:** R. W. Hauck, H. P. Emslander

**Affiliations:** 1 Medizinische Klinik und Poliklinik der Technischen Universität Pneumologie München Germany; 2 Medizinische Klinik und Poliklinik Pneumologie Ismaninger Strasse 22 München D-81675 Germany

## Abstract

Lung cancer is a leading cancer site in men and women with a high incidence and mortality rate. Most patients are diagnosed when the disease has already spread. An early, detection and immediate and accurate histological or cytological diagnosis are essential for a hopeful outcome. In most patients, bronchoscopy is the method of choice in establishing a suspected lung neoplasm. With the rigid and flexible method, two complementary techniques are available. The methods bear a very low mortality rate if sufficient monitoring and resuscitative instrumentation is available. Rigid bronchoscopy offers the possibility of obtaining large biopsy specimens from the tumorous tissue and provides an effective tool in the control of major haemorrhage. However, it cannot be used for the inspection of further peripherally located parts of the bronchial system and needs general anaesthesia. In contrast, the flexible method can be quickly and readily performed at practically any location using portable equipment. Bronchi can be inspected up to the 8th order and with bronchial washing, forceps biopsy, brush biopsy and fluorescence bronchoscopy techniques with a high diagnostic yield are available. This holds true, especially if these sampling techniques are used as complementary methods.


*Diagnostic and Therapeutic Endoscopy*, Vol. 1, pp. 9–18
Reprints available directly from the publisher
Photocopying permitted by license only

© 1994 Harwood Academic Publishers GmbH
Printed in Malaysia

### Bronchoscopy of Lung Cancer

R. W. HAUCK and H. P. EMSLANDER

*1. Medizinische Klinik und Poliklinik der Technischen Universität, Pneumologie, München, FRG*

*(Received December 9, 1993; in final form December 9, 1993)*

Lung cancer is a leading cancer site in men and women with a high incidence and mortality rate. Most patients are diagnosed when the disease has already spread. An early detection and immediate and accurate histological or cytological diagnosis are essential for a hopeful outcome. In most patients, bronchoscopy is the method of choice in establishing a suspected lung neoplasm. With the rigid and flexible method, two complementary techniques are available. The methods bear a very low mortality rate if sufficient monitoring and resuscitative instrumentation is available. Rigid bronchoscopy offers the possibility of obtaining large biopsy specimens from the tumorous tissue and provides an effective tool in the control of major haemorrhage. However, it cannot be used for the inspection of further peripherally located parts of the bronchial system and needs general anaesthesia. In contrast, the flexible method can be quickly and readily performed at practically any location using portable equipment. Bronchi can be inspected up to the 8th order and with bronchial washing, forceps biopsy, brush biopsy and fluorescence bronchoscopy techniques with a high diagnostic yield are available. This holds true, especially if these sampling techniques are used as complementary methods.

**KEY WORDS:** bronchoscopy, lung cancer, biopsy techniques

### INTRODUCTION

In the majority of countries throughout the world, lung cancer is the most common and lethal form of malignancy. Within the European community, this type of tumor is the leading cancer site for males and, in the last few years, this tumor type has even surpassed breast cancer as the most frequent cause of cancer death in women (Bunn, 1991; Ginsberg *et al.*, 1993).

The incidence of lung cancer currently exceeds 70 per 100,000 men (Ginsberg *et al.*, 1993). In the European community, approximately 157,000 cases of lung cancer are newly diagnosed each year, 23,000 of which are in women, leading to a death toll of approximately 135,000. This number accounts for around 30% of all cancer deaths.

While rates of increase for other types of tumors have remained stable over the past 20 years, those for lung cancer mortality have actually climbed (Boring *et al.*, 1991). This increase has been calculated to stand at 10–15% every 5 years among males and is closely linked to prevailing smoking habits (Saccommano *et al.*, 1976). During the 1980s however, the rate of increase was not as rapid as was observed in former years, thereby reflecting a trend towards cessation of smoking (National Center for Health Statistics, 1991).

There are four major histological subtypes for lung cancer: squamous cell carcinoma, adenocarcinoma, and large cell carcinoma, which are referred as non-small cell carcinoma (NSCLC) and, the group of small cell carcinoma (SCLC). Over the last decade, there has been an increased tendency for adenocarcinomas, especially of the bronchoalveolar type (Gazdar and Linnoila, 1988), along with a decrease of the proportion of squamous cell cancers (Valaitis *et al.*, 1981; Vincent *et al.*, 1977; Wu *et al.*, 1986). The presence of adeno- and bronchoalveolar carcinoma, as well as, in part, the very heterogenous group of large cell carcinoma, is observed more often within the peripheral parts of the lung, while squamous cell and small cell carcinoma occur more centrally.

Mean 5-year survival for persons with lung cancer stands at only 13% (Ginsberg *et al.*, 1993). This is reflected by the fact that the majority of patients are diagnosed, once the disease has already spread to distant sites. Therefore, the only chance of hopeful survival is an optimal management

Address for correspondence: Dr. Rainer W. Hauck, 1. Medizinische Klinik und Poliklinik, Pneumologie, Ismaninger Strasse 22, D-81675 München, F.R.G.

of the diagnostic tools with detection of the tumor before the disease has reached an incurable stage. This aim can be realized through immediate and accurate histological or cytological diagnosis combined with a complete staging. This is particularly relevant owing to the significant differences in survival at different stages of disease and to the differenciated therapeutic modalities currently available. In one-half of the cases, if a roentgenologically occult cancer is suspected, it will be visible at a first bronchoscopic inspection (Edell and Cortese, 1989). Those patients with an early stage, radiographically occult tumor do especially benefit from this approach demonstrated by the overall 5-year survival rate of 90% (Feruson, 1990).

Following careful collection of patient history and physical examination, the first reasonable step in the sequence of diagnostic evaluation is a chest roentgenogram which provides the most preliminary of investigations, should the diagnosis of lung cancer be suspected (Feruson, 1990). Usually a "T"-classification of the tumor, according to the TNM-staging protocol, can be assigned and some information pertaining to nodal enlargement ("N") is obtained. Other relevant findings include pleural effusion, diaphragmatic elevation and lobar atelectasis or dystelectasis. The next noninvasive staging assessment is computerized tomography of the chest, which allows assessment of tumor margins and dimensions. It can also help in distinguishing malignant from nonmalignant lung and lymph nodes. Additionally, infiltration of the pleura may be observed. Sputum cytology can be of diagnostic value especially for centrally located tumors (Feruson, 1990).

Most often, the information derived from these noninvasive diagnostic steps provides the basis for a subsequent invasive procedure. These are either methods which are performed via endoscopy or over a transthoracic route. For peripheral lung tumors or pleurally based lesions the transthoracic route carries a higher diagnostic yield in that it is performed using bronchoscopical techniques. The golden standard for transthoracic biopsy is achieved using open lung biopsy, with a diagnostic yield of approximately 95% and low mortality (Venn *et al.*, 1985). Within the last century, percutaneous needle biopsy has been well established for the diagnosis of mid-and peripheral lung lesions but the good diagnostic yield (~80%) is associated with a high rate of pneumothoraces (Berquist *et al.*, 1980; Poe *et al.*, 1984). Recently, less invasive techniques, i.e., thoracoscopic and videothoracoscopic lung biopsies, are available to harvest large tissue specimens by means of minimal surgical techniques (Feruson, 1990; Boutin *et al.*, 1982; Bensard *et al.*, 1993). However, for endobronchial lesions, atelectasis, or lobar consolidation, such methods cannot be recommended for use as a diagnostic procedure (Westcott, 1980).

In parallel to optimized surgical techniques for harvesting lung parenchyma, the endoscopical methods for diagnosis of lung cancer have steadily improved over the last 25 years. Today, fiberbronchoscopy is the method of choice in establishing a suspected lung neoplasm in most patients.

### BRONCHOSCOPY—TECHNIQUES AND INSTRUMENTS

Two complementary techniques are available for bronchoscopy, each offering different merits and disadvantages (Table 1). Respiratory physicians usually prefer the flexible bronchoscopy, while thoracic surgeons tend to prefer the rigid technique. If one is familiar with both techniques, it is also possible to pass the flexible bronchoscope through the rigid instrument which allows the advantageous use of both instruments. In the hands of experienced and skilled operators, both methods are safe and associated with minimal complications. The mortality is very low and stands at 0.002–0.3% (Surrat *et al.*, 1976).

### Premedication

Premedication consists of the subcutaneous application of 0.5–1 mg of atropine, 30 minutes prior to the investigation which reduces mucus secretion and counteracts vagal stimulation. Fifty to 100 mg of meperidine can be admixed to the atropine, thereby providing a reliable antitussive and analgetic effect (Nakhosteen *et al.*, 1989). Additionally, local anaesthesia must be applied, for example with lignocaine. This can be performed by means of a hand atomizer, with the aid of small swabs soaked in anesthetic solution or by drug nebulization. When the bronchoscope has been inserted to the larynx, the vocal cords, trachea and bronchi become anaesthetized by injecting lignocaine

**Table 1** Comparison of properties from rigid and flexible bronchoscopy

| | *Rigid bronchoscopy* | *Flexible bronchoscopy* |
|---|---|---|
| Local anaesthesia | (+) | + |
| General anaesthesia | + | (+) |
| Clarity of view | +++ | +++ |
| Mucosal aspect | +++ | ++ |
| Inspection of upper lobes | (+) | +++ |
| Visible generations of bronchi | –2nd | –10th |
| Sampling of proximal airways | ++ | +++ |
| Sampling of mid and peripheral airways | (+) | +++ |
| Size of biopsy specimen | +++ | ++ |
| Removal of foreign bodies | +++ | ++ |
| Management of major haemorrhage | +++ | + |
| Risk for morbidity | + | (+) |
| Risk for mortality | (+) | (+) |
| Costs | ++ | + |

with a syringe through the working channel of the scope (DeKock, 1977; Brown, 1984).

### Monitoring

Cardiac rhythm should be continuously monitored by ECG during and shortly following the investigation along with blood pressure in all patients undergoing bronchoscopy. Additionally, the arterial oxygen saturation must be assessed, usually by means of noninvasive devices (Fulkerson *et al.*, 1993). Two methods are available with ear oximetry and transcutaneous measurement of the oxygen saturation in the capillary blood, which provide accurate data for this parameter (Saunders *et al.*, 1976; McDowell and Thiele, 1980). Supplemental oxygen of 1–2 l/min can be administered to patients throughout the investigation, and regularly to those with an arterial $paO_2$ of less than 70 mm Hg (Fulkerson *et al.*, 1993). Because bronchoscopy is an invasive method and can lead to situations of emergency, the direct availability of resuscitative instrumentation is obligatory.

### Rigid Bronchoscopy

Rigid bronchoscopy is usually performed under general anesthesia which is often combined with intravenous muscle relaxation. Oxygen-Venturi systems are often used to provide a sufficient gas exchange during the procedure (Sanders, 1967). Alternatively, good results for oxygenation are obtained through the use of high-frequency positive-pressure ventilation (Eriksson and Sjöstrand, 1977; Munteanu *et al.*, 1986). The bronchoscope is a straight tube which is positioned into the tracheobronchial tree of the supine patient. Scopes are available in different calibers ranging from 3 to 8.5 mm. The right-sized scope is one which just passes the vocal cords with ease. The scope usually incorporates a seperate channel for the application of oxygen and anesthetics as well as a second one through which catheters may be passed. For the bronchoscopes, telescopes 30 cm in length with different angles of vision (0°, 30°, 90°) are available which can be positioned through the observers port. These freely interchangeable viewing telescopes can be connected with a cold light source. With the telescopes, which provide excellent optics, inspection at least to the level of segmental and first subsegmental bronchi is possible. The rigid bronchoscope offers the possibility to observe the trachea and the proximal parts of the main bronchi with a direct naked eye view and without any distortion evoked by an optical device. This provides the most natural and realistic investigation of structural deformities, abnormal rigidity and alteration to the mucosa.

In diagnosing lung cancer, the rigid bronchoscope offers the possibility to obtain a large biopsy specimen from the tumorous tissue by means of specially designed forceps's devices, which can be passed through the observers port. Through this large channel, a potential hemorrhage can be easily controlled by intense suction, insertion of a distensible balloon or a rigid tube, by electrocautery or cryocoagulation.

It is a disadvantage, however, that inspection of further peripherally located parts of the bronchial system is impossible with the rigid scope and that long and flexible instruments cannot be used in the sampling of peripheral lesions. Moreover, this investigation cannot be implemented as a bedsite method and usually requires general anesthesia that requires a well-trained and experienced nurse and technical staff, leading to an increase of costs for the investigation.

### Flexible Bronchoscopy

Since its introduction by Ikeda in 1968 (Ikeda *et al.*, 1968), the flexible bronchofibrescope has led to great progress in the practice of bronchoscopy. This is due to several advantages in comparison to the rigid method. Depending upon the situation, the scope can be introduced orally or via the transnasal route. Beside this, it is possible in intensive care situations to insert the instrument through a endotracheal or tracheostomy tube. Bronchoscopy with a flexible instrument can be performed under local anesthesia with some sedation. In cases of respiratory impaired patients, the method can even be carried out under general anesthesia with artificial ventilation (see above). Flexible bronchoscopy can be quickly and readily performed at practically any location using portable equipment and, if required, by a physician without additional staff.

Over the past 20 years, the quality and technical details of flexible scopes have been constantly improved upon. Today, bronchofiberscopes are available with a length ranging from 70–80 cm, thereby allowing sufficient access to even the very distal bronchi. The outer diameter of the instruments varies from 3.6 to 6.4 mm. In relation to this size, sucking channels from 1.2 to 3.2 mm are internally provided. Additionally, ultrathin instruments are available with an outer diameter of 2 mm, and no working channel (i.e. Olympus XPF-25 A-5 or XPF-18 N-5). These instruments can be passed through the sucking channel of a normal-sized scope. This "mother-baby scope" technique allows inspection of the bronchi down to the 7th or 8th order. The normal sized bronchoscope contains a flexible bundle of approximately 14,000 glass fibers, each with a diameter of 10 to 15 μm. Together with the lens system at the proximal and distal end of the bronchoscope, an image is received which is different from that naturally obtained by a direct inspection with the rigid scope. The angle of view varies from 90 to 120° depending upon the instrument. The magnification, evoked by the lens system, amounts to 2.5–3. The distal end of the bronchoscope can be angulated, thereby allowing entrance to the lobar or segmental bronchi. Based upon the type of scope used, a forward angulation from 160–180° and a backward flexion of 100 to 130° is possible. In contrast to gastroscopes, the tip of the bronchoscope can only be moved in a single plane, steered by a fine wire system. Together with rotation of the whole instrument a view in any direction is allowed.

### Bronchoscopic Inspection

Bronchoscopy permits direct inspection of the tracheobronchial tree and facilitates diagnosis of lung cancer along with the guided biopsy of suspective tissue structures. The method is essential in evaluating patients for subsequent surgery in that the anatomical behavior of the tumor is directly scrutinized.

During inspection for tissue or structures suspected for carcinoma, it is important to explore the entire tracheobronchial tree at all of its accessible parts as thoroughly as possible. If secretions are present, they must be carefully removed to provide an unrestricted view of all the luminal structures. Hereby, damage to the bronchial mucosa must be avoided in that this can lead to bleeding which would once again obscure the view. Tumors or enlarged lymph nodes can be indicated by three types of direct tumor signs: 1. exophytic intraluminal growth with stenosis of the inner surface (Fig. 1); 2. infiltration of the bronchial wall (Fig. 2); 3. distortion and compression of normal anatomic structures (Fig. 3).

The most intraluminal tumors are visible as irregular structures replacing the normal mucosa. Tumor extent can vary from a localized mucosa alteration to a solid tumor, resulting in a partial to total occlusion the bronchial lumen (Fig. 1). These tissues frequently consist of greater necrotic parts and their color may impone from red to eggshell and whitish. Those visible tumors are mostly primary lung cancers or carcinoid tumors, whereas metastases from other primary sites are commonly located in the lung parenchyma. Benign endoluminal tumors usually have a characteristically hard consistency and an intact covering mucosa.

Lung cancer which has less intensively infiltrated the bronchial wall may just impone either on a narrowed lumen of the bronchi, on an altered structure of the bronchial mucosa or by teleangiectatic blood vessels. Additionally, those areas become more suspicious, due to

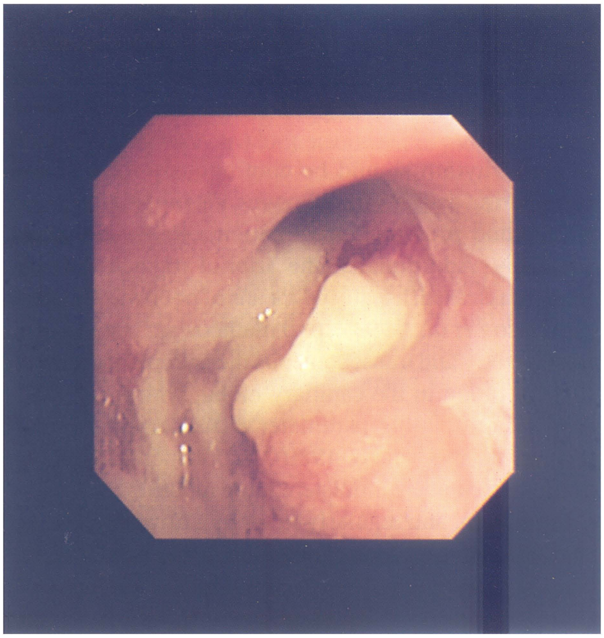

**Figure 1** Exophytic carcinoma (histology: squamous cell carcinoma) of the right main bronchus seen through a video-optic chip bronchoscope (Olympus, BF-XT 30).

an increased fragility of the blood vessels following contact with the bronchoscope. Often, enlarged and penetrated lymph nodes, as well as centrally located and ulcerated tumors may be identified which may have led to dislocation and compression of the normal anatomic structures of the bronchial tree.

Beside these direct tumor signs, the bronchial system must be inspected as well for such indirect evidences of malignancy as (Stradling, 1976): 1. vocal chord paralysis; 2. thickened and immobile bifurcation; 3. rigid and fixed

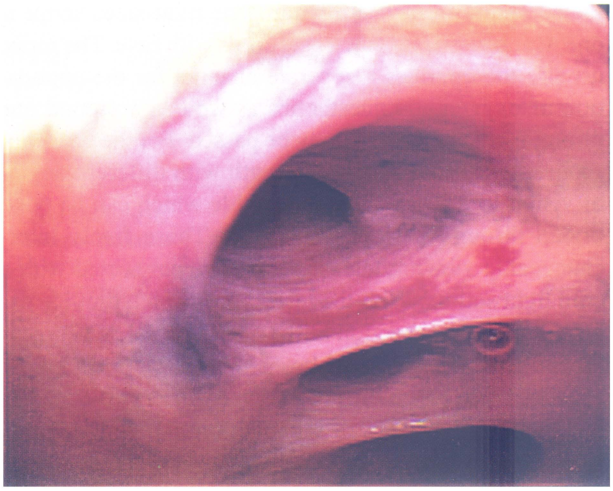

**Figure 2** Intrinsic carcinoma of the lungs with bulging (↓) the bronchus wall close to ostiums of the right lower lobe seen through a video-optic chip bronchoscope (Pentax, EB 2000).

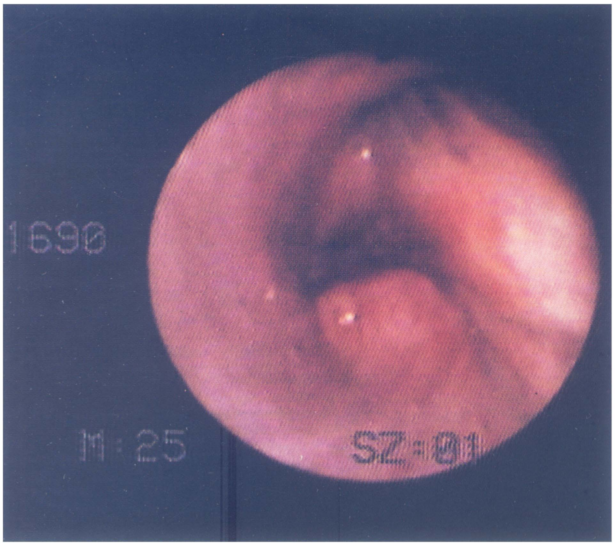

**Figure 3** Extrinsic carcinoma of the lungs (histology: small cell carcinoma) leading to distorsion and compression of anatomic structures in its neighbourhood seen through a fiberoptic bronchoscope (Olympus, BF-XT 30).

bronchial structures at breathing or coughing maneuvers; 4. deviation or cessation of mucosal corrugations; 5. impairment of fluid drainage into the periphery visible at the bronchial ostium.

Hoarseness in association with lung tumors is mostly due to paralysis of the vocal chords. This indirectly points to an infiltration of the laryngeal nerve at its intrathoracic course by tumor tissue or enlarged lymph nodes. Within the trachea and the proximal bronchial system tumor masses can lead, by pressure from the external site, either to bulging of the inner bronchial lumen, to distortion of the airways (Fig. 3) or to widening and fixation of the bicurcation of the trachea (Fig. 2). If it is impossible to visualize the tumor directly, the detection of rigid, immobile or hyperplastic airway structures can provide reliable aid in the decision as to where biopsy samples should be taken from. Tumor masses growing close to the airway wall often disturb the normal aspect of mucosal corrugations which must be documented with thorough bronchoscopical inspection. In addition, the presence of an invisible obstruction within the distal parts of the bronchi can be suspected if anesthetics or lavage fluids demonstrate a loss of their run off to the lung periphery at the level of the carina.

Fluorescence bronchoscopy can be potentially useful in the imaging and procuring of malignant tissue (Lam *et al.*, 1993), especially in the diagnosis of occult lung cancer, early cancer, or early invasive squamous cell carcinoma, stages in which therapy often provides an excellent prognosis (Feruson, 1990; Hayata *et al.*, 1984; Kato *et al.*, 1986). Fluorescent compounds such as hematoporphyrin derivate ($H_pD$) or its purified form dihematoporphyrin ether (DHE) are preferentially retained by malignant cells and by tissue with cellular atypia. Because those cells concentrate these compounds in contrast to nonmalignant cells, they can be identified with several bronchoscopic detection systems through a higher emission of red fluorescence following excitation by ultraviolet light (Edell and Cortese, 1989; Lam *et al.*, 1993; Kato *et al.*, 1984). Very recently it could be shown that comparable results can be obtained without the use of fluorescent compounds (Edell and Cortese, 1989) through the use of spectroscopy during bronchoscopic investigation with a multichannel analyzer and a helium cadmium laser.

### Possible Complications

Lignocain, which is widely used for local anesthesia, can lead to toxic side effects. Adverse reactions may occur as broncho- or laryngospasm (Ruffles and Gayres, 1987), loss of consciousness, hypotension, or in the form of convulsions. Therefore, it is recommended not to exceed a total dose of 500 mg or 24 ml of the 2% solution (DeKock, 1977; Grimes and Cates, 1976). Use of the 2% solution seems to carry a lower risk for side effects, yet an equal effectiveness, than the 4% solution (Brown, 1984). To prevent the induction of bronchospasm in asthmatics, premedication with 50 mg of methylprednisolone is recommended followed by albuterol administered as an intrabronchial instillation (Nakhosteen, 1978). The latter can also be used to treat attacks of severe coughing induced by bronchoscopy. Because the arterial partial pressure of oxygen can decrease by about 20 mm Hg during bronchoscopy (Dubrawsky *et al.*, 1975; Emslander *et al.*, 1980), patients should be routinously supplied with supplemental oxygen during the procedure to avoid hypoxemia. Rarely cardiac arrythmias occur, mostly in patients with coronary heart disease. In 10–20% of the patients (Fulkerson *et al.*, 1993; Eriksson and Sjöstrand, 1977), especially after bronchoalveolar lavage, fever up to 39° can occur, which usually relieves within 8 hours without specific therapy (Table 2).

**Table 2**

| *Complications from Bronchoscopy* |
|---|
| • Drug reaction |
| • Bronchospasm |
| • Laryngospasm |
| • Convulsive cough |
| • Hypoxemia |
| • Cardiac arrythmia |
| • Fever |

Performed by a skilled physician, bronchoscopy is seldom if ever contraindicated, especially when a suspected lung tumor must be verified. Relative contraindications exist in extremely impaired or intensely dyspnoeic patients and in patients with recent myocardial infarction (DeKock, 1977).

### Bronchoscopical Sampling

Each patient suspected of having a pulmonary tumor based upon the results obtained from noninvasive procedures should undergo cytological or histological confirmation of the diagnosis (Feruson, 1990). Different methods of biopsy for the harvesting of tumor tissue are available (Fig. 4). The method with the highest diagnostic yield should be selected, taking into careful consideration, however, the patient's physical condition (Table 3).

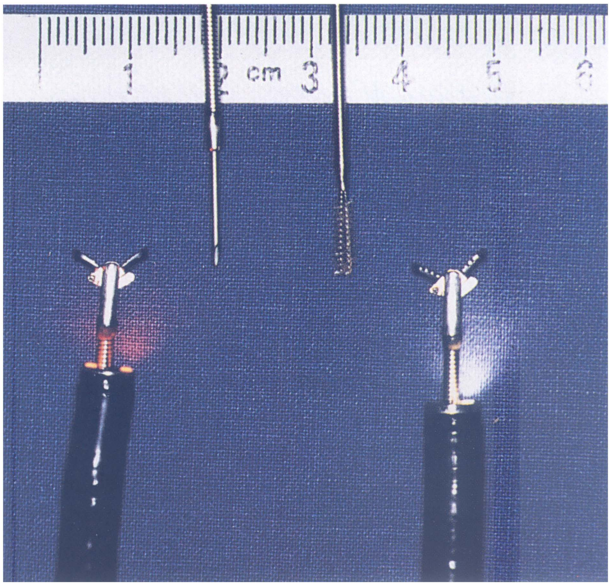

**Figure 4** Close-up photograph of the tip of different types of biopsy instruments for use with the fiberoptic bronchoscope. At the top, a 20 gauge needle and a brush, at the bottom a cup and an alligator forceps, each inserted in the working channel of a flexible bronchoscope.

### Bronchoalveolar Lavage (BAL)

In the past, BAL was widely used as a research tool which provided access to cells and proteins from the lung periphery. However, previous reports have demonstrated that BAL can be useful for the diagnosis of primary lung cancer and metastatic malignancies (Rennard, 1992; Pirozynski, 1992; Levy *et al.*, 1988), especially in cases where the lesions are not endoscopically visible (Table 3). Here, the BAL has a diagnostic yield of about 65% (Levy *et al.*, 1988; Cortese *et al.*, 1979), which in part is dependent upon the size (diameter >3 cm) and type of the underlying tumor. Higher diagnostic yields are obtained for alveolar cell lung cancer and for adenocarcinomas (Pirozynski, 1992; Linder and Rennard, 1988; Lundgren *et al.*, 1983). Complications with BAL are nearly negligible. Minor side effects after BAL consist in some cases, of transient fever as well as in a short-term decline in lung function testing. Rarely, pneumothorax can occur as well as slight bleeding, but these complications are below the frequency seen with other biopsy methods (Rennard, 1992). In visible tumors, bioptic techniques yield superior results in affirming the diagnosis however, in peripherally located cancers cytological techniques are superior (Kvale *et al.*, 1976). The diagnostic yield of the BAL can be increased by performing the lavage following all other sampling methods and rises with the size of the suspected lesion (Pirozynski, 1992). False-positive results are not

**Table 3** Diagnostic yields and complication rates of different bronchoscopical biopsy techniques.

| *Method* | *Diagnostic Yield* | *Pneumothorax* | *Haemorrhage* | *Morbidity* | *Mortality* |
|---|---|---|---|---|---|
| Endobronchial biopsy: | 76–96%[50;52;69] | ∅ | 0[57] | 0.45%[58] | 0.01–0.04%[58;59] |
| Bronchoalveolar lavage: | | | | | |
| Visible lesion | 79%[69] | <0.1%[39] | <0.1%[39] | <2%[39] | <0.1[39]% |
| Non-visible lesion | 43–65%[40,60] | | | | |
| Brushing technique: | | | | | |
| Visible lesion | 92%[69] | <1%[70] | 0[70] | 0 | 0 |
| Nonvisible lesion | 25–67%[40;50;51;60;61] | | | | |
| Transbronchial needle biopsy: | 33–76%[51;63;65;67] | <1%[51;67;70] | 0[51;70] | 0[51] | 0[61;67] |
| Transbronchial forceps biopsy: | | | | | |
| Lesion <4 cm | 58%[61] | 0.01–5.5%[58;59;61;70] | 4%[68] | 1.4–7%[61;68] | 0.12%[59] |
| Lesion >4 cm | 81%[61] | | | | |

reported (DeGracia *et al.*, 1993), whereas cell-type differences between the diagnosis of malignancy obtained from BAL samples compared to histological biopsies can occur in about 20–40% (Pirozynski, 1992; Linder *et al.*, 1987). Additionally, dysplastic alterations of the harvested cells in patients with pneumonia, viral infections, tracheostomy, radiotherapy or chemotherapy must be taken into consideration while it can make it difficult to distinguish these cell from those with malignant alterations (Johnston *et al.*, 1991).

The BAL is carried out by the administration of a total volume of 20–200 ml (alliquots of 20 ml) of warmed 0.9% NaCl with a hand-held syringe through the working channel of the bronchoscope into the region of interest. If only a defined suspicious segment of the lung shall be lavaged, it is possible to instill the washing solution to this area under the guidance of a small ERPC-catheter. Following gentle aspiration, the returned samples are pooled and processed. A recovery of 30% or more from the injected volume can be considered as representative (Pirozynski, 1992; DeGracia *et al.*, 1993; Klech and Pohl, 1989). Saline washings should be dispatched to the laboratory immediately in that cell morphology deteriorates rapidly upon storage (Brown, 1984).

### Brushing Technique

Similarly to the BAL, brush biopsy is nearly free of relevant complications (Schenk *et al.*, 1986). The method is used for the diagnosis of both visible tumors and lesions which are not visible endoscopically as well for tumors located in parts of the tracheobronchial tree which cannot be reached by forceps biopsy. The method offers a diagnostic yield (Table 3) which for visible tumors, amounts to approximately 90% (Haponik *et al.*, 1988) and for nonvisible lesions to approximately 25–67% (Pirozynski, 1992; Mak *et al.*, 1990; Shure, 1983; Cortese *et al.*, 1979; Ellis, 1975). Either "blind", by brushing the suspected lung segment after determination by posteroanterior and lateral chest X-rays (Mak *et al.*, 1990) or under fluoroscopic guidance, this method is reliable but can be optimized by the additional use of other biopsy techniques. Together with transbronchial biopsy, the yield can increase to around 95% for visible and to 75% for nonvisible tumors (Mak *et al.*, 1990; Shure, 1983; Popovich *et al.*, 1982; Cortese *et al.*, 1979). However, even then, the failure rate for diagnosing lung cancer amounts to 25–35%.

Several types of flexible brushes are available. Naked brushes are just mounted at the tip of a leading catheter, protected brushes are covered by a thin plastic tube which can be removed immediately before the sample shall be obtained. The catheter is inserted through the working channel of the bronchofiberscope and positioned at the region of interest. To harvest cells, the brush can be slightly moved in an up-down motion along the tissue and rotated about. The brushings are then smeared on three or four slides and fixed in 95% alcohol until investigation.

### Endobronchial Biopsy

Endobronchial biopsy is the golden standard for sampling endoscopically visible tumors and tumorous alterations of tracheal and bronchial mucosa (Table 3). The method provides a yield of positive diagnosis of malignancy in about 96% (Mak *et al.*, 1990; Popovich *et al.*, 1982; Haponik *et al.*, 1988) and allows a high rate (80%) of accuracy in predicting cell type (Bayne *et al.*, 1979). If the tumor is well visible, the yield is greater than in "blind" biopsy. In the latter case, a positive diagnosis constitutes about 45% of the cases but only with an accuracy of 60% (Bayne *et al.*, 1979). Often biopsies from even "normal mucosa" can be useful in order to exclude submucosal infiltrative carcinoma. In selecting the site for taking a biopsy necrotic or hypervascular areas should be avoided. Usually endobronchial forceps biopsy carries a low risk for haemorrhage except for patients with clotting abnormalities, uremia or pulmonary hypertension (Zavala *et al.*, 1988).

Three major groups of forceps are available in varying sizes (3–5 mm): Cup forceps, fenestrated forceps, alligator forceps, and forceps with a inside thorn. It is not necessary to harvest a large specimen, it is of more importance to select from the right place (Stradling, 1976). So far, it is not clarified what is the optimal number of biopsies to secure an adequate tissue diagnosis. However, 3–5 biopsies from central visible tumors (Popovich *et al.*, 1982; Kvale, 1978; Fechner *et al.*, 1977) and up to 10 biopsies from peripheral carcinomas are generally recommended (Popovich *et al.*, 1982). A single biopsy should be regarded as technically inadequate if malignant cells could not be harvested (Pirozynski, 1992) (Table 4).

### Transbronchial Biopsy

Lung cancer which is beyond the visible bronchi can be diagnosed by means of transbronchial biopsy. A well-circumscribed peripheral lung lesion biopsy is carried out

**Table 4**

| *Influences on Diagnostic Yield of Lung Biopies* |
|---|
| • Location of the lesion: (proximal/distal) |
| • Site of the tumor (intra-/extraluminal) |
| • Size of lesion |
| • Number of biopsies |
| • Combination of sampling techniques |

under fluoroscopic guidance. The closed forceps is passed to the tumor and following injection of 3–5 ml of epinephrine (1:10,000) the biopsy specimens are taken. After taking the biopsy, it is removed from the forceps and placed into 10% formol saline until its processing. For diffuse processes *i.e.*, lymphangiosis or alveolar cell carcinoma, the forceps are either engaged to the most suspicious area previously demonstrated on chest X-ray, or on computed tomography, or preferably placed to the lateral segment (S9) of the lower lobe. Usually 4–6 specimens are taken (Zavala *et al.*, 1988). To avoid a possible bilateral air leak, biopsies are only allowed from one lung side. The overall mortality rate of transbronchial biopsy amounts to 0.12%, the rate of major complications to 2.7% (Simpson *et al.*, 1983). Transbronchial biopsy carries an incidence of pneumothorax from 0.01 to 5.5% (Table 3) depending upon the site of the lesion from where the biopsy is taken and whether or not radiological screening is used for the procedure (Hanson and Collins, 1990; Simpson *et al.*, 1983; Ellis, 1975; Haponik *et al.*, 1988). To prevent bleeding complications, patients should provide normal prothrombin time, activated partial thromboplastin time, platelet count and should not suffer from clotting abnormalities. At a peripheral biopsy, the risk for major bleeding is lower compared to biopsies from more central parts of the lungs because of its smaller sized blood vessels (Hanson and Collins, 1990). The overall diagnostic yield of transbronchial biopsy amounts to 67–80%. For peripheral lesions below 4 cm in diameter, the rate is 58%; for larger tumors, it rises to 81% (Cortese *et al.*, 1979; Ellis, 1975).

### Transbronchial Needle Aspiration

Needle aspiration via the flexible bronchoscope is mainly applied in diagnosing bronchogenic carcinoma metastasising to hilar or mediastinal nodes (Wang, 1983). In a series of 363 patients with newly diagnosed cancer of the lung, this method yielded positive mediastinal aspirates in 34% (Harrow *et al.*, 1989). Due to its central origin, the rate for diagnosing small-cell carcinoma is twice as high as for non-small cell cancers. Interestingly, this method also provides positive results for N2 nodes (ipsilateral mediastinal or subcarinal lymph nodes) if no abnormality of the chest X-ray (46%) or tracheobronchial tree (38%) to this point is visible (Harrow *et al.*, 1985). The diagnostic yield for detecting N2 disease stands at 77% and offers a specificity close to 100% (Wang *et al.*, 1983; Schenk *et al.*, 1986).

Along with this, transbronchial needle aspiration is commonly used for the sampling of extrabronchial and peripheral tumors as well as for lesions compressing the bronchus leading to an avoidance of forceps placement. Additionally, needle aspiration can provide sufficient specimens from grossly necrotic tumors and can be considered for use in the diagnosis of highly vascularized masses (Table 5). The size of the lung mass directly influences the diagnostic yield of the procedure (Shure and Fedullo, 1983) with a significant increase in positive results for lesions exceeding 2 cm in size (33% versus 76%). If used in combination with bronchial wash and brush for lesions exceeding 2 cm, transbronchial needle aspiration appears to be superior to transbronchial biopsy (Shure and Fedullo, 1983). The method is safe and very rarely associated with complications such as severe bleedings (Harrow *et al.*, 1989; Shure and Fedullo, 1983) (Table 3).

Aspiration needles usually have a size of 20–23 gauge by 1 cm. They are attached to a Teflon tubing and housed in a metal sheath, in which the needle can be retracted for the passage through the working channel of the bronchoscope. At the end of the catheter, a syringe can be attached to create suction for aspiration. Usually, two or three punctures are performed at each biopy site. To avoid contamination of malignant cells, needle biopy should always precede other diagnostic procedures (Zavala *et al.*, 1988). Harvested cells should be collected as previously described.

### Summary

Lung cancer has advanced to a position as the leading malignant disease in most countries of the world. To provide an optimal chance for a hopeful outcome, it is essential to assure a firm tissue diagnosis for planning the therapeutic approach. Depending upon the location and spread of the cancer, more or less invasive diagnostic procedures are available. In recent years bronchoscopy has become the most commonly used tool in establishing suspicious lesions, evident on chest X-ray. About two-thirds of all tumorous alterations can be seen by endoscopy directly or indirectly and can be biopsied to affirm a tissue diagnosis. With the rigid and flexible bronchoscopy, two complementary endoscopical techniques are available. Due to its easy availability, lack of need for general anesthesia and the possibility to inspect bronchi even to the 7th or 8th order, flexible bronchofiberscopes are presently most commonly used. With visible lesions, forceps biopsy, bronchial washing and brush biopsy should be performed,

**Table 5**

| *Indications for Transbronchial Needle Aspiration* |
|---|
| • Mucosal infiltration |
| • Mediastinal and extrabronchial lymph node enlargement |
| • Extrinsic tumor with compression of the bronchial tree |
| • Necrotic tumor mass |
| • Vascular, carcinoid tumor |

if possible, as complementary methods to raise the diagnostic yield. For the diagnosis of nonvisible, more peripherally growing lung cancer, transbronchial biopsies, brushings, needle aspiration under fluoroscopic guidance, and selective washings are the most reliable methods. In cases of *carcinoma* in situ fluorescence bronchoscopy can assist to localize such lesions. For each kind of lesion, the combination of different sampling methods increase the probability of obtaining a firm histological and cytological diagnosis, without a relevant increase of side effects.

Along with these excellent diagnostic properties, bronchoscopy provides the possibility for the administration of a variety of treatments suitable for the palliation of lung neoplasms. The available methods include stent implantation, laser therapy, cryotherapy, fulguration, injection of locally acting cytotoxic drugs, insertion of radioactive gold grains, endoscopic radiation techniques, and photodynamic therapy. However, this field of interventional bronchology is the subject of further elaboration in another paper.

In conclusion, different locations of pulmonary pathologies require a differenciated strategy of securing sufficient material to make a definitive histopathological diagnosis. Based upon the advanced technique of the flexible bronchoscope, well developed biopsy equipment, easy availability and even the possibility to perform this method on an outpatient basis, this investigation has become the workhorse in establishing lung cancer.

### REFERENCES

Baglin J. Y., Danel C., Lacronique J., Jaubert F., Chrétien J.: Interest of bronchoalveolar lavage (BAL) in the diagnosis of lung tumors with normal fiberoptic bronchoscopic examination. International conference on bronchoalveolar lavage, Bethesda, 1984

Bayne C. R., Stovin P. G. I., Barker V., McVittie S., Stark J. E.: Diagnostic accuracy of cytology and biopsy in primary bronchial carcinoma. *Thorax*, 1979;34:294–299

Bensard D. D., McIntyre R. C., Waring B. J., Simon J. S.: Comparison of video thoracoscopic lung biopsy to open lung biopsy in the diagnosis of interstitial lung disease. *Chest*, 1993;103:765–770

Berquist T. H., Bailey P. B., Cortese D. A., *et al.*: Transthoracic needle biopsy. *Mayo Clin Proc*, 1980;55:475–481

Boring C. C., Squire T. S., Tong T.: Cancer statistics. *CA*, 1991; 41:19d

Boutin C., Viallat J. R., Cargnino P., Rey F.: Thoracoscopic lung biopsy. *Chest*, 1982;82:44–49

Brown M. M.: Bronchoscopy with rigid and flexible instruments. In: Bates M. (ed.): *Bronchial carcinoma*, First Edition, Springer Verlag Berlin, Heidelberg, New York, Tokyo, 1984

Bunn P. A. (ed.): Current topics in lung cancer. Springer Verlag, Berlin, Heidelberg, New York, 1991

Cortese D. A., McDougall J. C.: Biopsy and brushing of peripheral lung cancer with fluoroscopic guidance. *Chest*, 1979;75:141–145

DeGracia J., Bravo C., Miravitlles M., *et al.*: Diagnostic value of bronchoalveolar lavage in peripheral lung cancer. *Am Rev Respir Dis*, 1993;147:649–652

DeKock M. A. (ed.): Dynamic bronchoscopy, First Edition, Springer Verlag Berlin Heidelberg, 1977

Dubrawsky C., Awe R. J., Jenkins D. E.: The effect of bronchofiberscopy examination on oxygenation status. *Chest*, 1975;67:137–140

Edell E. S., Cortese D. A.: Bronchoscopic localization and treatment of occult lung cancer. *Chest*, 1989;96:919–924

Ellis J. H.: Transbronchial biopsy via the fiberoptic bronchoscope. *Chest*, 1975;68:524–32

Emslander H. P., Cyran J., Krüger R., *et al.*: Diagnostische und therapeutische Möglichkeiten der Fiberbronchoskopie in der internistischen Intensivmedizin. *Intensivmed*, 1980;17:10–13

Eriksson I., Sjöstrand U.: Experimental and clinical evaluation of high-frequency positive pressure ventilation (HFPPV) and the pneumatic valve principle in bronchoscopy under general anesthesia. *Acta Anaesthesiol Scand*, 1977;64(Suppl.):83

Fechner R. E., Greenberg S. D., Wilson R. K., Stevens S. P. M.: Evaluation of transbronchial biopsy of the lung. *Am J Clin Pathol*, 1977;68:17–20

Feruson M. K.: Diagnosing and staging of non-small cell lung cancer. *Hematol-Oncol-Clin-North-Am*, 1990;4:1053–1068

Fulkerson W. J., Tapson V. F.: Bronchoscopy. In: Holland J. F. (ed.): Cancer Medicine, Third Edition, Lea and Febiger, Malvern, Pennsylvania, 1993

Gazdar A. F., Linnoila I.: The pathology of lung cancer—changing concepts and newer diagnostic techniques. *Semin Oncol*, 1988;15:215–225

Ginsberg R. J., Kris M. G., Armstrong J. G.: Cancer of the lung. In: DeVita V. T., Hellman S., Rosenberg S. A. (eds.): Cancer: Principles and practise of Oncology, Fourth Edition. J. B. Lippincott Co., Philadelphia, 1993

Grimes D. A., Cates W.: Deaths from paracervical anesthesia used for first trimester abortion. *N Engl J Med*, 1976;295:1397–1399

Hanson P., Collins J.: Bronchoscopy and lavage. In: Brewis R. A. L., Gibson G. J., Geddes D. M. (eds.): *Respiratory medicine*, First Edition, Baillière Tindall, W. B. Saunders, London, 1990

Haponik E. F., Kvale P., Wang K. P.: Bronchoscopy and related procedures. In: Fishman A. P. (ed.): *Pulmonary Diseases and Disorders*, First Edition, McGraw-Hill Inc., 1988

Harrow E. M., Oldenburg F. A., Lingenfelter M. S., Smith A. M.:Transbronchial needle aspiration in clinical practise. *Chest*, 1989;96:1268–72

Harrow E. M., Oldenburg F. A., Smith M. A.: Transbronchial needle aspiration in clinical practice. *Thorax*, 1985;40:756–59

Hayata Y., Kato H., Konaka C., *et al.*: Photoradiation therapy with hematoporphyrin derivative in early and stage I lung cancer. *Chest*, 1984;86:169–177

Ikeda S., Yanai N., Ishikana S.: Flexible bronchofiberscope. *Keio J Med*, 1968;17:1–16

Johnston W. W., Elson C. E.: Cytology of other body sites. Respiratory tract. In: Bibbo M. (ed.): *Comprehensive Cytopathology*, W.B. Saunders, Philadelphia, 1991

Kato H., Aizawa K., Ono J. *et al.*: Clinical measurement of tumor fluorescence using a new diagnostic system with hematoporphyrin derivate, laser photoradiation, in a spectroscope. *Laser Surg Med*, 1984;4:49–58

Kato H., Konaka C., Kawate N., Shinohara H., Kinoshita K., *et al.*: Five-year disease-free survival of a lung cancer patient treated only by photodynamic therapy. *Chest*, 1986;90:768–770

Klech H., Pohl W.: Technical recommendations and guidelines for bronchoalveolar lavage (BAL). *Eur Respir J*, 1989;2:561–585

Kvale P. A., Bode F. R., Kini S.: Diagnostic accuracy in lung cancer. Comparison of techniques used in association with flexible fiberoptic bronchoscopy. *Chest*, 1976;69:752–757

Kvale P. A.: Collection and preparation of bronchoscopic specimens. *Chest*, 1978;73S:707–712

Lam S., MacAulay C., Palcic B.: Detection and localization of early lung cancer by imaging techniques. *Chest*, 1993;103:12S–14S

Levy H., Horak D. A., Lewis M. I.: The value of bronchial washings and bronchoalveolar lavage in the diagnosis of lymphangitic carcinomatosis. *Chest*, 1988;94:1028–30

Linder J., Radio S. J., Robbins R. A., Ghafouri M., Rennard S. I.: Bronchoalveolar lavage in the cytologic diagnosis of carcinoma of the lung. *Acta Cytol*, 1987;31:756–757

Linder J., Rennard S. I.: *Bronchoalveolar Lavage*. ASCP Press, Chicago, 1988

Lundgren R., Bergman F., Angstrom T.: Comparison of transbronchial fine needle aspiration biopsy, bronchial washings, brush biopsy and forceps biopsy in the diagnosis of lung cancer. *Eur J Respir Dis*, 1983;64:378–385

Mak V. H. F., Johnston I. D. A., Hetzel M. R., *et al.*: Value of washings and brushings at fiberoptic bronchoscopy in the diagnosis of lung cancer. *Thorax*, 1990;45:373–376

Martini N., McCormick M.: Assessment of endoscopically visible bronchial carcinomas. *Chest*, 1978;73(Suppl.):718–720

McDowell J. W., Thiele W. H.: Usefullness of the transcutaneous $pO_2$ monitor during exercise testing in adults. *Chest*, 1980;78:853–855

Munteanu J., Müller C., Emslander H. P., Heinl K. W., Hinke K., Daum S.: Hochfrequente Jet-Ventilation in der Bronchoskopie. Atemw-Lungenkrkh, 1986;7:314–317

Nakhosteen J. A., Niederle N., Zavala D. C. (eds.): Atlas und Lehrbuch der Bronchoskopie. Springer Verlag Berlin Heidelberg New York, Second Edition, 1989

Nakhosteen J. A.: Fiberoptic bronchoscopy in reversible airways disease. In: Tokito K. (ed.): Bronchoscopy, WCB, Tokyo, 1978

National Center for Health Statistics. Health: United States 1990. US Department of Health and Human Services Publication (PHS)91–1232. Washington, DC: US Government Printing Office, 1991

Pirozynski M.: Bronchoalveolar lavage in the diagnosis of peripheral, primary lung cancer. *Chest*, 1992;102(2):372–74

Poe R. H., Kallay M. C., Wicks C. M., Odoroff C. L.: Predicting risk of pneumothorax in needle biopsy of the lung. *Chest*, 1984;85:232–235

Popovich J., Kvale P. A., Eichenhorn M. S. Radke J. R., Ohorodnik J. M., Fine T.: Diagnostic accuracy of multiple biopsies from flexible fibreoptic bronchoscopy. *Am Rev Respir Dis*, 1982;125:521–523

Rennard S. I.: Bronchoalveolar lavage in the diagnosis of lung cancer. *Chest*, 1992;102(2):331–2

Ruffles S. P., Gayres J. G.: Fatal bronchospasm after topical lignocaine before bronchoscopy. *Br Med J*, 1987;294:1658–1659

Saccomanno G., Archer V. E., Saunders R. P., *et al.*: Early indices of cancer risk among uranium miners with reference to modifying factors. *Ann NY Acad Sci*, 1976;271:377–383

Sanders R. D.: Two ventilating attachments for bronchoscopes. *Delaware Med J*, 1967;39:170–175

Saunders N. A., Powles A. C. P., Rebuck A. S.: Ear oximetry: Acurracy and practicability in the assessment of arterial oxygenation. *Am Rev Respir Dis*, 1976;113:745–749

Schenk D. A., Bower J. H., Bryan C. L., Currie R. B., Spence T. H., Duncan C. A. *et al.*: Transbronchial needle aspiration in staging of bronchogenic carcinoma. *Am Rev Respir Dis*, 1986;134:147–148

Shure D., Fedullo P. F.: Transbronchial needle aspiration of peripheral masses. *Am Rev Respir Dis*, 1983;128:1090–1092

Shure D., Fedullo P. S.: Transbronchial needle aspiration of peripheral masses. *Am Rev Respir Dis*, 1983;128:1090–1092

Simpson F. G., Arnold A., Purvis A., *et al.*: Postal survey of bronchoscopic practice by physicians in the United Kingdom. *Thorax*, 1983;41:311–317

Stradling P. (ed.): Diagnostic bronchoscopy, Third Edition, Longman Inc., New York, 1976

Surrat P. M., Smiddy J. F., Gruber B.: Death and complications associated with fiberoptic bronchoscopy. *Chest*, 1976;69:747–51

Valaitis J., Warren S., Gambel D.: Increasing incidence of adenocarcinoma of the lung. *Cancer*, 1981;47:1042–1046

Venn G. E., Kay P. H., Midwood C. J., *et al.*: Open lung biopsy in patients with diffuse pulmonary shadowing. *Thorax*, 1985;40:931–935

Vincent R. G., Pickren J. W., Lane W. W., *et al.*: The changing histopathology of lung cancer. A review of 1682 cases. *Cancer*, 1977;39:1647–1655

Wang K. P., Brower R., Haponik E. K., Siegelman S.: Flexible transbronchial needle aspiration for staging of bronchogenic carcinoma. *Chest*, 1983;84:571–76

Wang K. P., Haponik E. F., Britt E. J., Khouri N., Erozan Y.: Transbronchial needle aspiration of localized pulmonary lesions: results in 422 patients. *Radiology*, 1980;137:31–35

Wang K. P., Terry P. B.: Transbronchial needle aspiration in the diagnosis and staging of bronchogenic carcinoma. *Am Rev Respir Dis*, 1983;127:344–47

Westcott J. L.: Direct percutaneous needle aspiration of localized pulmonary lesions: results in 422 patients. *Radiology*, 1980;137:31–35

Wu A. H., Henderson B. E., Thomas D. C., *et al.*: Secular trends in histologic types of lung cancer. *J Natl Cancer Inst*, 1986;77:53–56

Zavala D. C.: Bronchoscopy, lung biopsy, and other procedures. In: Murray J. F., Nadel J. A. (eds.): *Textbook of Respiratory Medicine*, First Edition, W. B. Saunders, Philadelphia, 1988

